# Nanoparticles Isolated From Porcine Bone Soup Ameliorated Dextran Sulfate Sodium-Induced Colitis and Regulated Gut Microbiota in Mice

**DOI:** 10.3389/fnut.2022.821404

**Published:** 2022-03-29

**Authors:** Huiqin Wang, Jin Huang, Yanan Ding, Jianwu Zhou, Guanzhen Gao, Huan Han, Jingru Zhou, Lijing Ke, Pingfan Rao, Tianbao Chen, Longxin Zhang

**Affiliations:** ^1^Food Nutrition Science Centre, School of Food Science and Biotechnology, Zhejiang Gongshang University, Hangzhou, China; ^2^School of Pharmacy, Queen’s University Belfast, Belfast, United Kingdom; ^3^Fujian Provincial Maternity and Children’s Hospital, Affiliated Hospital of Fujian Medical University, Fuzhou, China

**Keywords:** bone soup, nanoparticles, ulcerative colitis, gut microbiota, inflammation, dextran sulfate sodium

## Abstract

Daily foods contain a great number of self-assembled nanoparticles (NPs) which were incidentally produced during food processing. These food incidental NPs can directly access the human gastrointestinal tract in high frequency and large quantities. Limited reports were focused on whether and how these food incidental NPs affected the gastrointestinal tissues and gut microbiota. In the present study, bone soup and its NPs both significantly ameliorated colitis symptoms in dextran sulfate sodium (DSS)-induced mice and inhibited the release of pro-inflammatory cytokines. They also restored intestinal microbiota dysbiosis by improving the diversity and richness of intestinal microbiota and regulating community composition, such as a remarkable increase in *Muribaculaceae*, *Alistipes*, and *Alloprevotella*, and a decrease in *Helicobacter*. Moreover, the correlation analysis showed that pro-inflammatory cytokines were negatively correlated with *Muribaculaceae*, *Alloprevotella*, and *Alistipes*, but positively correlated with *Helicobacter*. These findings suggest that the food incidental NPs can influence human health through regulating the inflammation of the gastrointestinal tissues and the gut microbiota.

## Introduction

In the past decades, nanoparticles (NPs) have been increasingly used in food processing and food packaging for improving the quality of food, extending the shelf-life, enhancing the uptake, absorption, and bioavailability of food nutrients, etc (1, 2). These engineered NPs could have direct access to the human gastrointestinal tract (GIT) along with the food (3) and subsequently affect the gut microbiota and the GIT itself, such as GIT inflammation and redox homeostasis (4). Several studies revealed that these NPs, such as TiO_2_ (5), SiO_2_ (6), CuO (7), Ag (8), could induce intestinal inflammation and disruption of gut microbiota. On the contrary, some engineered NPs, e.g., Ce oxide (9), ZnO (10), and Ag (11) exerted an anti-inflammatory activity. The underlying mechanisms of the “nanoparticle-GIT” interaction were not fully understood, thus warranting further studies.

Apart from the engineered NPs in the food system, daily foods contained abundant amounts of incidental NPs, too, which were the assemblies of polar or non-polar food components derived by physical and chemical forces during the food processing (heating, cooling, and emulsification, etc.) (12). For instance, the proteoglycan–lipid NPs with an average diameter of 50–67 nm were generated during the heating processing of Freshwater Clams (*Corbicula fluminea* Muller) soup (12). Numerous spherical NPs were found in green tea infusions (13). A large number of self-assembly micro/nanoparticles containing docosahexaenoic acid (DHA) and eicosatetraenoic acid (EPA) have been observed in bigeye tuna (*Thunnus obesus*) head soup (14). Moreover, porcine bone soup, as a traditional nourishing food for preventing immune malfunction and inflammatory bowel disease (IBD) (15, 16), has been demonstrated to contain amounts of nanoscale colloidal particles with an average size of ca. 200 nm, and the formation of these incidental NPs has been investigated (17, 18). These food incidental NPs not only played a crucial role in the stability and texture characterization of food, but also made great contributions to the physiological effects of food due to the unique physicochemical properties at nanoscale. Similar to the engineered NPs, these food incidental NPs may directly encounter cells in human GIT and alter the intestinal microenvironment. To date, however, little has been known on these incidental food NPs and their biological impacts on the gut.

As we previously reported, the spherical NPs mainly consisted of lipids and proteins with an average size of ca. 200 nm and ζ-potential of ca. −15 mV derived from porcine bone soup were isolated and directly interacted with macrophages with preventing cells from peroxyl radical-induced membrane hyperpolarization, mitochondrial malfunction, and phagocytosis suppression (18). Moreover, these NPs derived from the porcine bone soup attenuated oxidative stress-induced intestinal barrier injury in the Caco-2 cell monolayer model (17). In this study, we set off to evaluate the alleviative effects of NPs derived from porcine bone soup on dextran sulfate sodium (DSS) induced colitis in mice, by modulating intestinal inflammation and gut microbiota.

## Materials and Methods

### Preparation of Nanoparticles Derived From Porcine Bone Soup

Fresh porcine bones (Landrace pigs, *Sus scrofa*) were obtained from the fresh local market. The bone soup was prepared, and the NPs were isolated according to our previous reports (17–19). Briefly, 1 kg of fresh porcine bones was cooked with deionized water (1:3, w/v) for 3 h in a boiling water bath after removing the blood residues. Filtered through four layers of cotton gauze to remove the solid residues and the soup was centrifuged at 400 *g* for 10 min. The supernatant of bone soup was collected and named as BS. Then BS was applied to a pre-equilibrated size-exclusive chromatographic column. The fractions with strong light scattering intensity were collected and dialyzed against water, and the obtained NPs samples were freeze-dried and designated as BSNPs. Before the animal experiment, BSNPs were dissolved with deionized water to the same derived count rate as that of BS.

### Animal Experiments

The 5-week-old specific pathogen-free (SPF) female BALB/c mice were supplied by Zhejiang Center of Laboratory Animals (Hangzhou, China). The mice were housed under the standard conditions (21 ± 1°C, 55.5 ± 5% relative humidity) with a controlled light–dark cycle of 12/12 h. The mice were fed on standard chow and distilled water *ad libitum*. After 1 week of adaptive feeding, the mice were randomly divided into five groups with six mice each. Acute colitis in BALB/c mice was established according to the previous study (20). The groups were as follows: (1) Control group, mice were fed with standard chow and distilled water *ad libitum*, daily administered normal saline (50 ml/kg/day) by oral gavage for 7 days. (2) DSS group, provided free access to 5% DSS (36–50 kDa; MP Biomedicals, Irvine, CA, United States) in distilled water and daily administered normal saline (50 ml/kg/day) by oral gavage for 7 days. (3) Sulfasalazine (SASP) group, provided free access to 5% DSS in distilled water and daily administered SASP (250 mg/kg, Shanghai Zhongxi Sanwei Pharmaceutical Co., Ltd., Shanghai, China) by oral gavage for 7 days. (4) The BS group provided free access to 5% DSS in distilled water and administered BS at 50 ml/kg/day by oral gavage for 7 days. (5) BSNPs group provided free access to 5% DSS in distilled water and administered BSNPs at 50 ml/kg/day by oral gavage for 7 days. On the 8th day, mice were sacrificed by cervical dislocation. The fecal samples were collected for 16S ribosomal RNA (16S rRNA) analysis. The colon samples were excised quickly and rinsed with ice-cold 0.9% NaCl. Their length and weight were subsequently measured. At last, colons were divided into three parts for histopathological analysis and real-time polymerase chain reaction (PCR).

All animal experiments were conducted according to the NIH Guidance for the care and use of laboratory animals and approved by the Animal Care and Welfare Committee of Zhejiang Center of Laboratory Animals, China.

### Disease Activity Index

The severity of colitis was evaluated daily according to the DAI scoring system as described previously (21), such as body weight loss, stool consistency, and gross rectal bleeding.

### Histological Analysis

For histological evaluation, hematoxylin and eosin (H&E) stained colon tissues were performed, and histological changes were evaluated by two independent investigators in a blinded manner according to a scoring system as described previously (22). The sum of each score reflected the colonic histological damage.

### RNA Extract and Real-Time PCR

The mRNA expression of inflammatory cytokines, such as interleukin 6 (IL-6), interleukin 1 beta (IL-1β), and tumor necrosis factor-alpha (TNF-α), were determined in ileum mucosa by real-time PCR. Total RNA was extracted from the colon tissue using with miRNeasy Mini Kit (QIAGEN, 217004, Hilden, North Rhine-Westphalia, Germany), its concentration and purity were measured using a NanoDrop 2000 spectrophotometer (Thermo Fisher Scientific, Waltham, MA, United States). cDNA was synthesized using RevertAid First Strand cDNA Synthesis Kit (Thermo Fisher Scientific, K1622, Waltham, MA, United States). The real-time PCR was conducted using Platinum SYBR Green qPCR SuperMix-UDG with ROX (Invitrogen, 11744-500, Carlsbad, CA, United States); the reaction solution contained 10 μl of 2 × SYBR Green, 1 μl of each primer (10 μM), 1 μl of reverse transcription product, and 8 μl of RNase/DNase-free water (total volume 20 μl). Glyceraldehyde 3-phosphate dehydrogenase (GAPDH) was used as an internal reference gene to normalize target gene transcript levels and the primers sequences of target genes (Sangon Biotech (Shanghai) Co., Shanghai, China) are presented in Table 1. The reaction conditions were as follows: 2 min at 95°C, 40 cycles for 15 s at 95°C, 30 s at 60°C, 30 s at 72°C. All the results were normalized to the internal reference gene: GAPDH. The relative expression of mRNA level was calculated with the 2^–ΔΔ*Ct*^ method.

**TABLE 1:** GenBank accession numbers, sequences of forward and reverse primers, and fragment sizes used for real-time PCR.

Target	GenBank number	Primer sequence	Size, bp
*TNF*-α	NM_013693.3	F:5′ TTGTCTACTCCCAGGTTCTCT3′	107
		R: 5′ GAGGTTGACTTTCTCCTGGTATG3′	
*IL-6*	NM_031168.2	F:5′ CTTCCATCCAGTTGCCTTCT3′	134
		R:5′ CTCCGACTTGTGAAGTGGTATAG3′	
*IL-1*β	NM_008361.4	F:5′ CCACCTCAATGGACAGAATATCA3′	96
		R:5′ CCCAAGGCCACAGGTATTT3′	
*GAPDH*	NM_001289726.1	F:5′ AACAGCAACTCCCACTCTTC3′	111
		R:5′ CCTGTTGCTGTAGCCGTATT3′	

### 16S rRNA Analysis

The gut microbiota composition of mice feces was determined by 16S rRNA gene amplification. Briefly, MagPure Stool DNA KF Kit B (Magen Biotechnology Co., Guangzhou, China) was used to extract genomic DNA from feces. DNA concentration and integrity were measured by a NanoDrop 2000 spectrophotometer (Thermo Fisher Scientific, Waltham, MA, United States) and agarose gel electrophoresis, respectively. The 16S rRNA gene V3–V4 region was amplified from the genomic DNA in a 25 μl reaction using the universal bacterial primers: (343F, 5′- TACGGRAGGCAGCAG-3′, and 798R, 5′- AGGGTATCTAATCCT-3′). The PCR products were purified with Agencourt AMPure XP beads (Beckman Coulter Co., Brea, CA, United States) and quantified by using a Qubit dsDNA assay kit (Thermo Fisher Scientific, Waltham, MA, United States). Sequencing was performed on an Illumina NovaSeq 6000 with two paired-end read cycles of 250 bases each (Illumina Inc., San Diego, CA, United States; OE Biotech Co., Ltd., Shanghai, China).

The Trimmomatic software was used to analyze the raw data. After trimming, operational taxonomic units (OTUs) were generated by using VSEARCH 2.7.1 with a 97% similarity cutoff. The representative read of each OTU was selected by using the Quantitative Insights into Microbial Ecology (QIIME) package. All representative reads were annotated and blasted against the Silva database (Version 132) using the RDP classifier (confidence threshold was 70%). The microbial richness and diversity in fecal content samples were estimated using the alpha diversity that includes Chao1 index, Observed species index, Simpson index, and Shannon index. The UniFrac distance matrix performed by QIIME software was used for the unweighted UniFrac Principal coordinates analysis (PCoA), Non-metric multidimensional scaling (NMDS) analysis, and phylogenetic tree construction. Linear discriminant analysis effect size (LEfSe) based linear discriminant analysis (LDA) and cladogram were generated to assess differentially abundant microbial taxa. The 16S rRNA gene amplicon sequencing and analysis were conducted by OE Biotech Co., Ltd. (Shanghai, China).

### Statistical Analysis

Data were expressed as mean ± standard error of the mean (SEM) and performed with GraphPad Prism 7.0 (GraphPad Software, Inc., San Diego, CA, United States). The significant differences were analyzed using one-way analysis of variance (ANOVA) followed by Tukey’s multiple comparisons in multiple groups. Correlations were analyzed by using Spearman’s correlation analysis. The value of *p* < 0.05 was considered to be statistically significant.

## Results

### Bone Soup Nanoparticles Alleviated the Symptoms of Dextran Sulfate Sodium-Induced Colitis in Mice

**FIGURE 1:**
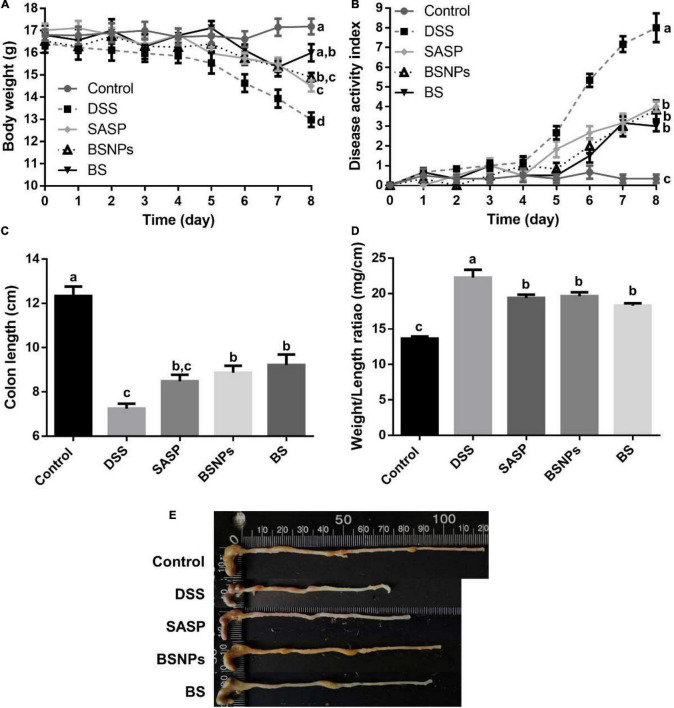
Effect of BS or BSNPs treatment on clinical symptoms of dextran sulfate sodium (DSS)-induced colitis. **(A)** Bodyweight measurement. **(B)** Disease activity index. **(C)** Colon length of mice. **(D)** Colon weight/length ratio. **(E)** Representative images of the colon from a different group. Values were expressed as the mean ± SEM (*n* = 6). Means without a common letter are significantly different at *p* < 0.05 using one-way ANOVA followed by Tukey’s test for *post hoc* analysis.

The influences of the BS or its BSNPs on the symptoms of DSS-induced colitis were investigated. As shown in Figures 1A–E, the DSS-treated group showed a significant reduction in bodyweight, increase in DAI score, reduction of colon length, and increment of colon weight/length ratio compared with the healthy control group (*p* < 0.05). The BS or BSNPs treatment, similar to that of SASP, significantly alleviated the bodyweight loss, the DAI score, and increment in the colon length. A significant reduction of colon weight/length ratio was observed in the BS/BSNPs-treated mice compared with the DSS group (*p* < 0.05). Moreover, no significant difference was observed between BS and BSNPs treatment, implying that the alleviative effects of BS in DSS-induced colitis might be mainly attributed to its NP components.

### Bone Soup Nanoparticles Attenuated the Inflammation of Colonic Mucosa

**FIGURE 2:**
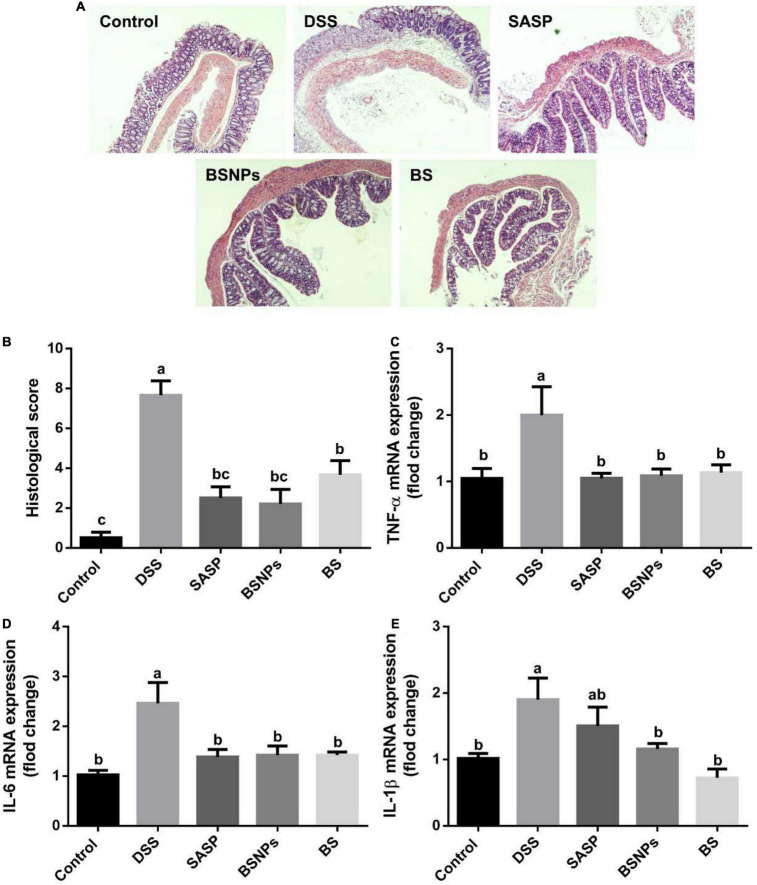
Effect of BS, BSNPs, and SASP treatment on histopathological alteration and inflammatory cytokines in the colonic mucosa of DSS-induced colitis. **(A)** Representative images of colonic segments. **(B)** Histological scores. **(C)** TNF-α expression. **(D)** Interleukin 6 (IL-6) expression. **(E)** Interleukin 1 β (IL-1β) expression. Values are expressed as the mean ± SEM (*n* = 6). Means without a common letter are significantly different at *p* < 0.05 using one-way ANOVA followed by Tukey’s test for *post hoc* analysis.

The histological changes of colonic mucosa were consistent with the general symptoms described above, determined with H&E staining. As shown in Figures 2A,B, the DSS treatment induced inflammatory cell infiltration and significantly elevated histological score (*p* < 0.05). The treatment of BS or BSNPs significantly inhibited the cell infiltration and histological score (*p* < 0.05).

In line with the histological changes, the pro-inflammatory cytokines (e.g., TNF-α, IL-6, and IL-1β) were boosted by DSS but downregulated by BS or BSNPs to an extent similar to that of SASP (Figures 2C–E), judging by quantitative real-time PCR determination of mRNA abundance of cytokines. The BS and BSNPs exhibited the same level of inhibition on colonic inflammation, elucidating the NPs are the principal active components of the bone soup in treating DSS-induced colitis.

### Bone Soup Nanoparticles Restored Gut Microbial Diversity

**FIGURE 3:**
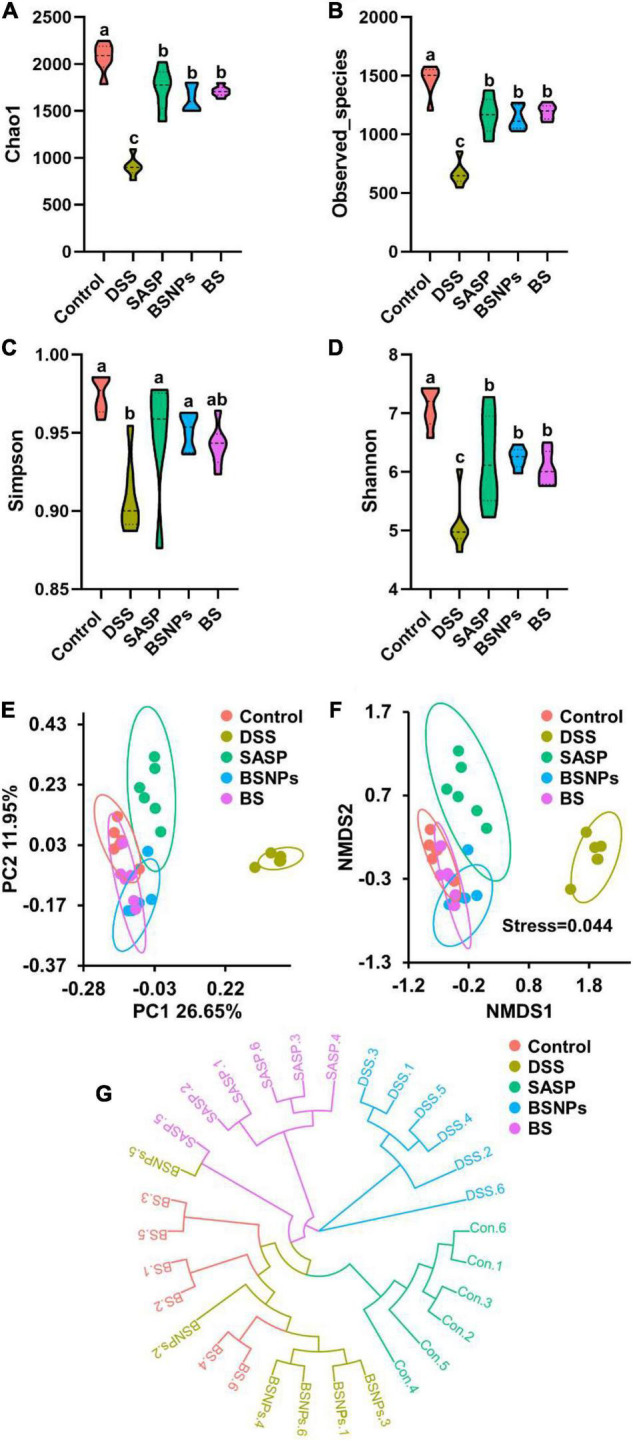
Analysis of the alpha diversity and beta diversity of the differential microbial community among the control group, DSS group, SASP, BS, and BSNPs-treated group. Violin plots showing Chao1 index **(A)**, Observed_species index **(B)**, Simpson index **(C)**, Shannon index **(D)**. **(E)** UniFrac distance of unweighted PCoA analysis, **(F)** UniFrac distance of unweighted NMDS analysis, **(G)** the unweighted pair-group technique with arithmetic mean (UPGMA) analysis the differences between samples. Means without a common letter are significantly different at *p* < 0.05 using one-way ANOVA followed by Tukey’s test for *post hoc* analysis.

The alpha diversity and beta diversity analysis of the gut microbial community in each group were assessed using a set of statistical analysis indexes. The gut microbial community richness index was calculated with Chao1 and Observed species index, while Shannon index and Simpson index were used to estimate the microbial diversity. As shown in Figures 3A–D, the Chao1 index, Observed species index, Shannon index, and Simpson index of the DSS group were found to be significantly lower than the normal mice, suggesting DSS reduced the microbial richness and diversity, disrupted the microbiota structure. The administration of either BS or BSNPs restored the microbial richness and diversity, in similar effectiveness with SASP. No significant difference was observed between the BS and BSNPs groups.

Besides, the PCoA of the microbial community showed that the microbial evolution of DSS-treated mice was vastly different from the normal mice, while the BS and BSNPs-treated mice were rather similar to the normal mice (Figure 3E). It is in line with the NMDS analysis (Figure 3F). In addition, the unweighted pair-group technique with arithmetic mean (UPGMA) was employed to construct a circular hierarchical clustering tree (Figure 3G), which indicated that the administration of BS, BSNPs, and SASP caused much fewer changes in microbial communities than that of DSS.

### Modulation of Gut Microbiota by Bone Soup Nanoparticles

**FIGURE 4:**
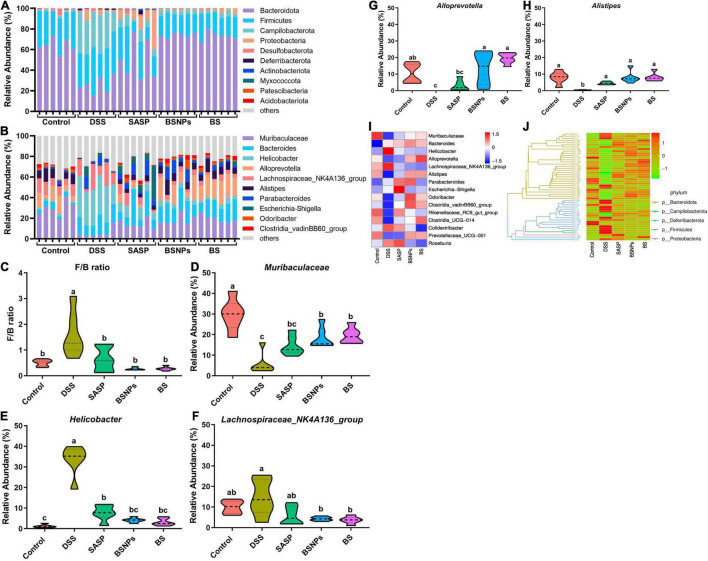
Analysis of the microbial community structure of each group. **(A)** Relative abundance of each sample at the phylum level. **(B)** Relative abundance of each sample at the genus level. Violin plots showing *Firmicutes* to *Bacteroidetes* ratio (F/B ratio) **(C)** of each group, and relative abundance of *Muribaculaceae* **(D)**, *Helicobacter* **(E)**, *Lachnospiraceae_NK4A136_group* **(F)**, *Alloprevotella* **(G)**, and *Alistipes* **(H)** at the genus levels. **(I)** Heatmap illustrating the relative abundance of the 15 most abundant bacterial genus. **(J)** Phylogenetic tree and heatmap showing relative abundances of operational taxonomic units (OTUs) together with a corresponding phylogenetic tree. The clustering branch represents different bacterial phyla. The abundance graph was shown on the right, which corresponds to the abundance of left OTUs in each sample. Means without a common letter are significantly different at *p* < 0.05 using one-way ANOVA followed by Tukey’s test for *post hoc* analysis.

The gut microbiota community structure distribution and relative abundance of taxa were analyzed. At the phylum level, the gut microbiota composition in each group was shown in Figure 4A. Compared to the normal mice, mice in the DSS group exhibited the higher relative abundances of *Campilobacterota* (33.43 vs. 1.26%) and lower relative abundances of *Bacteroidetes* (25.53 vs. 63.95%). The DSS induced an elevated *Firmicutes/Bacteroidetes* (F/B) ratio, too (*p* < 0.05, Figure 4C). The administration of BSNPs or BS significantly reversed these microbiota composition changes at the phylum level and resorted the F/B ratio to a normal level.

Consistently, the BSNPs or BS administration also restored the microflora balance at the genus level (Figure 4B), characterized by significantly increased *Muribaculaceae* (Figure 4D), *Alistipes* (Figure 4H), and *Alloprevotella* (Figure 4G), markedly decreased *Helicobacter* and *Lachnospiraceae_NK4A136_group* (Figures 4E,F) (*p* < 0.05). Furthermore, a heatmap based on the relative abundance of the top 15 abundant genera of bacteria, a phylogenetic tree, and a heatmap showing relative abundances of OTUs together with a corresponding phylogenetic tree were performed (Figures 4I,J). These results confirmed that BS or BSNPs treatment could counteract the DSS-induced changes in the gut microbiota.

**FIGURE 5:**
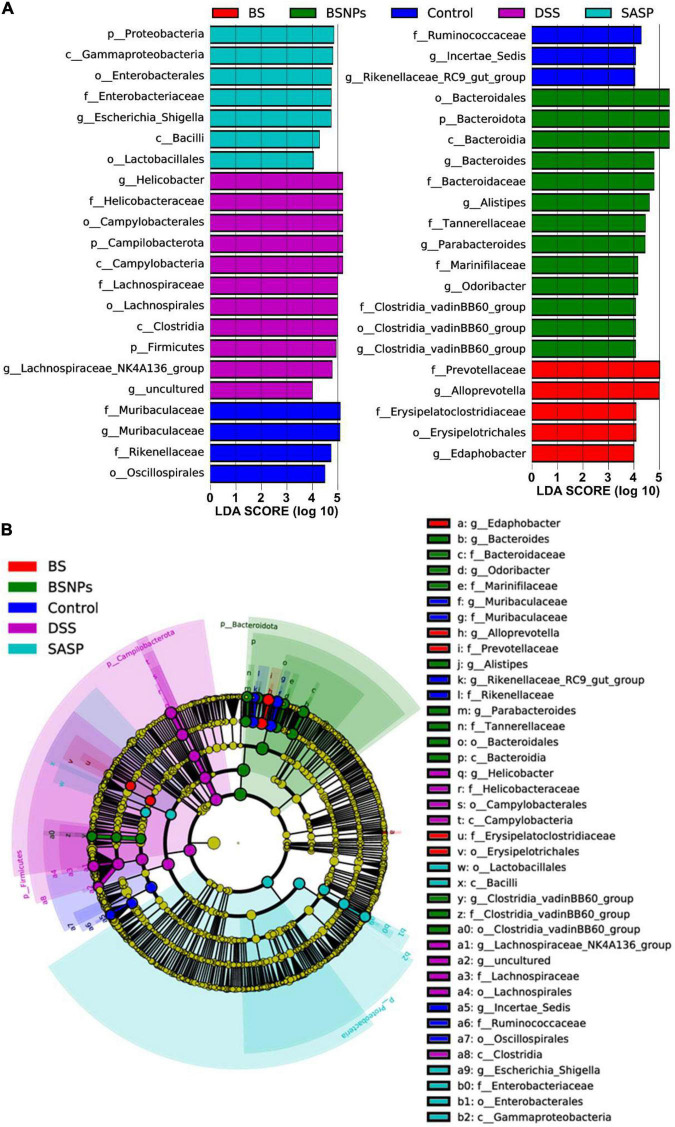
Linear discriminant analysis (LDA) and effect size (LEfSe) analysis of bacterial among the different groups. **(A)** LDA indicates the most differential abundant bacterial taxa in specific samples. **(B)** Cladogram illustrating highly abundant taxa across various treatments.

The LEfSe was performed to compare the difference in the abundance of gut microbiota compositions among different groups of mice (Figures 5A,B). From the phylum level to the genus level, there were 43 significant taxa in all groups. At the genus levels, *Lachnospiraceae_NK4A136_group* and *Helicobacter* were enriched in the DSS-treated mice, while 11 other taxa, such as *Muribaculaceae*, *Alloprevotella*, *Alistipes*, *Odoribacter*, *Clostridia_vadinBB60_group*, and *Rikenellaceae_RC9_gut_group*, were enriched in normal mice and SASP/BS/BSNPs-treated mice.

### Correlations Between the Expressions of Pro-inflammatory Cytokines With Microbiota

**FIGURE 6:**
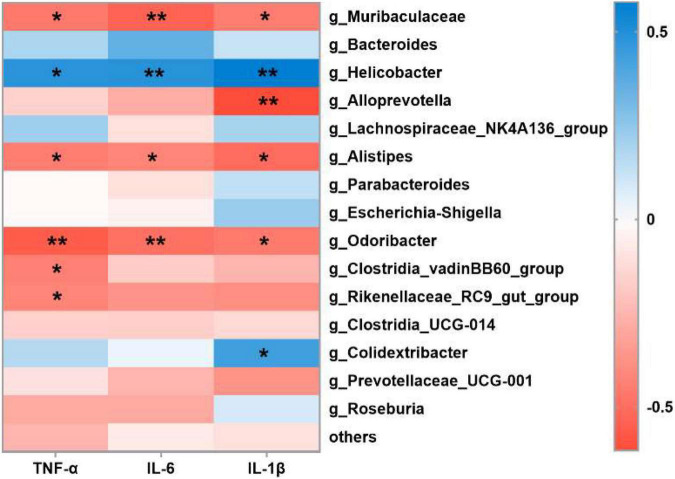
Interrelationship between gut microbiota and pro-inflammatory cytokines. Heatmap of Spearman’s correlation between gut bacteria (at the genus level) and pro-inflammatory cytokines. Significant difference determined at *p < 0.05, **p < 0.01.

The possible correlations between gut microbiota and pro-inflammatory cytokines were examined with Spearman’s correlation analyses (23). As shown in Figure 6, six bacteria were negatively correlated with pro-inflammatory cytokines (*p* < 0.05), such as *Muribaculaceae*, *Alloprevotella*, *Alistipes*, *Odoribacter*, *Clostridia_vadinBB60_group*, and *Rikenellaceae_RC9_gut_group*. Notably, these six bacteria were separately enriched in BSNPs, BS, and control group (Figure 5A), and *Muribaculaceae*, *Alloprevotella*, and *Alistipes* were significantly lower in the DSS group than other groups (Figures 4D,G,H). Furthermore, *Helicobacter and Colidextribacter* were significantly positively correlated with the pro-inflammatory cytokines (*p* < 0.05). Intriguingly, *Helicobacter* was enriched in DSS treatment (Figure 5A), and the treatment of BS, BSNPs, or SASP can significantly decrease the abundances of *Helicobacter* (Figure 4E). These results revealed that the anti-inflammatory effects of BSNPs and BS on DSS-induced colitis might be correlated to the increased abundance in *Muribaculaceae*, *Alloprevotella*, and *Alistipes*, and the decreased abundance in *Helicobacter* in the microbiota of mice.

## Discussion

The present study demonstrated that BS and BSNPs (the NPs isolated from bone soups) alleviated the pathological symptoms and inflammatory cytokines of DSS-induced colitis in mice. There was no significant difference between BS and BSNPs treatment, implying that BSNPs may exert protective effects against DSS-induced bowel disease in mice. BSNPs are mainly consisted of lipids and proteins, including collagen (18). It has been reported that the hybrid NPs of lipid–protein were resistant to hydrolysis of enzymes in saliva, stomach, and intestinal fluids and protected its inherent bioactive components from degradation (24, 25). The lipid–protein complex of BSNPs might make the particles resistant to gastrointestinal digestion, as was found in grapefruit-derived NPs (25). In our previous study, BSNPs have been demonstrated impressively high stability under harsh conditions of 4°C for 5 days (18). This indicated that BSNPs might as intact NP form to exert the anti-inflammatory effect in the gut.

Some inorganic NPs [such as Ce oxide (9), ZnO (10), and Ag (11)] could exhibit the therapeutic effects in the experimental colitis model *via* the anti-oxidative mechanism. Other NPs fabricated with natural biopolymers, such as chitosan and silk fibroin, acted as nanoscale drug delivery systems to alleviate ulcerative colitis (4, 26–28). The alleviative effects of BSNPs on intestinal inflammation might be attributed to its antioxidant activity and its constitutive collagen (17–19).

The cellular protective effects of BSNPs have been demonstrated in previous studies (17–19). According to the *in vitro* study, the BSNPs could be engulfed by oral and peritoneal macrophages, possess intracellular antioxidant activities, and suppress the membrane hyperpolarization induced by peroxyl radical on macrophage (18, 19). Such suppression could facilitate the anti-inflammatory effects (29). The healthier macrophages would promote the gut homeostasis and recovery of IBD (30). In addition, the disruption of the intestinal barrier is associated with disease of the GIT. Emerging evidence showed inadequate epithelial permeability resulted in IBD, IBS, UC, etc (31, 32). BSNPs could restore the intestinal barrier function of Caco-2 cells (17), which may also contribute to its alleviating effects on colitis.

Ulcerative colitis is accompanied by the dysbiosis of gut microbiota. Our results also revealed that BS or BSNPs could restore gut microbiota dysbiosis induced by the DSS. BS or BSNPs treatment increased the richness or diversity of gut microbial communities, which is badly damaged in IBD patients and DSS-induced colitis mice (33). The BS or BSNPs treatment markedly increased the abundance of *Muribaculaceae, Alistipes*, and *Alloprevotella*, while decreasing that of *Helicobacter.* The decrease of *Muribaculaceae* has been identified as the key to the development of colitis (34). *Alistipes* was reported to have protective effects against some diseases, including colitis (35). *Alloprevotella* was demonstrated to be lower in mice with DSS-induced colitis (36). *Helicobacter* was pathogens implicated in the formation of peptic ulcers and IBD (37). Moreover, the BS or BSNPs treatment decreased the F/B ratio. The ratio would be increased in colitis and other inflammatory diseases. A lower F/B ratio is connected to recovery or the health status (38, 39). Notably, our study demonstrated that *Muribaculaceae*, *Alloprevotella*, and *Alistipes* were negatively correlated with TNF-α, IL-6, and IL-1β. Some previous studies have shown that *Muribaculaceae*, *Alloprevotella*, and *Alistipes* were negatively correlated with inflammation, cause they promoted SCFA production and improved the intestinal tissue’s tolerance to the immune stimulations (34, 36, 40). In contrast, *Helicobacter* was positively correlated with three pro-inflammatory cytokines. Consistently, other reports have also described that *Helicobacter* genus could stimulate the immune response and cause the production of pro-inflammatory cytokines *via* activating proinflammatory nuclear factor kappa B (NF-κB) pathway (36, 41). These findings suggested that the protective effects of BSNPs on colitis might be attributed to its regulatory effects of gut microbiota correlated with immune responses and inflammation.

It has been reported that nanosized-food additives can bind to bacteria to form the NP-bacteria complex primarily *via* van der Waals attraction (42). This complex can induce the degradation of bacterial cell wall components, the alteration of metabolic pathways, oxidation of cellular components, and DNA damage, leading to the influence on the microbiome (43). In addition, naturally occurring NPs from beer could also bind to bacteria to form NP–bacteria complexation and then affect the microbiome (42).

However, it remains unknown whether the BSNPs could have the chance and ability to bind to the microorganisms in the alimentary tract and form a hybrid complex. The BSNPs may have been partially or completely digested before they reach the colon. The constitutive lipid and protein of BSNPs may also contribute to the bioactivities. The underlying mechanism of the interaction between NPs, their digestion products, and microbiota has not been fully understood and warrants further investigation.

## Conclusion

Altogether, our study demonstrated that the isolated NPs from bone soup showed a preventing effect against DSS-induced colitis. The treatment of BSNPs alleviated the severity of colitis with reduction of the release of inflammatory cytokines, restored gut microbiota dysbiosis by redirecting the bacterial shift to a normal state with increasing the richness or diversity of gut microbial communities and regulating community composition, such as a remarkable increase in *Muribaculaceae*, *Alistipes*, and *Alloprevotella*, and a decrease in *Helicobacter*. It indicated that the food-derived NPs could influence human health by regulating the inflammation of the gastrointestinal tissues and the gut microbiota.

## Data Availability Statement

The datasets presented in this study can be found in online repositories. The names of the repository/repositories and accession number(s) can be found below: https://www.ncbi.nlm.nih.gov/search/all/?term=PRJNA779815, Sequence Read Archive (SRA) database (PRJNA779815).

## Ethics Statement

The animal study was reviewed and approved by the Animal Care and Welfare Committee of Zhejiang Center of Laboratory Animals, China.

## Author Contributions

HW contributed to conceptualization, data curation, methodology, validation, formal analysis, and writing the original draft. JH and HH contributed to formal analysis, data curation, and methodology. YD and JRZ contributed to methodology, formal analysis, and investigation. JWZ contributed to formal analysis, supervision, and writing, reviewing, and editing the manuscript. GG contributed to conceptualization, methodology, supervision, investigation, validation, funding acquisition, project administration, and writing, reviewing, and editing the manuscript. LK contributed to formal analysis, project administration, and writing, reviewing, and editing the manuscript. PR contributed to conceptualization and writing, reviewing, and editing the manuscript. TC reviewed and edited the manuscript. LZ contributed to writing, reviewing, and editing the manuscript. All authors contributed to the article and approved the submitted version.

## Conflict of Interest

The authors declare that the research was conducted in the absence of any commercial or financial relationships that could be construed as a potential conflict of interest.

## Publisher’s Note

All claims expressed in this article are solely those of the authors and do not necessarily represent those of their affiliated organizations, or those of the publisher, the editors and the reviewers. Any product that may be evaluated in this article, or claim that may be made by its manufacturer, is not guaranteed or endorsed by the publisher.

## Abbreviations

NPs: nanoparticles
GIT: gastrointestinal tract
DSS: dextran sulfate sodium
DHA: docosahexaenoic acid
EPA: eicosatetraenoic acid
IBD: inflammatory bowel disease
BS: bone soup
BSNPs: bone soup nanoparticles
SASP: sulfasalazine
16S rRNA: 16S ribosomal RNA
DAI: disease activity index
GAPDH: glyceraldehyde 3-phosphate dehydrogenase
IL-6: interleukin 6
IL-1β: interleukin 1 beta
TNF-α: tumor necrosis factor alpha
PCoA: principal coordinates analysis
NMDS: non-metric multidimensional scaling
LEfSe: linear discriminant analysis effect size
OTUs: operational taxonomic units
LDA: linear discriminant analysis
NF-κB: nuclear factor kappa B.
